# The use of strategies from the social sciences to inform pipeline development programs for under-represented minority faculty and students in the health sciences

**DOI:** 10.1017/cts.2020.566

**Published:** 2020-12-14

**Authors:** Alicia K. Matthews, Paula Allen-Meares, Karriem Watson, Natasha Crooks, Ariel Smith, Alysha Hart, Mayra L. Estrella, Sage Kim

**Affiliations:** 1 Department of Population Health Nursing Science, College of Nursing, University of Illinois at Chicago, Chicago, IL, USA; 2 College of Medicine, University of Illinois at Chicago, Chicago, IL, USA; 3 Department of Community Health Sciences, School of Public Health, University of Illinois at Chicago, Chicago, IL, USA; 4 Department of Human Development Nursing Science, College of Nursing, University of Illinois at Chicago, Chicago, IL, USA; 5 Department of Health Policy and Administration, School of Public Health, University of Illinois at Chicago, Chicago, IL, USA

**Keywords:** Pipeline development programs, under-represented minorities, social sciences research, health sciences, recruitment and retention

## Abstract

Representation of under-represented minority (URM) faculty in the health sciences disciplines is persistently low relative to both national and student population demographics. Although some progress has been made through nationally funded pipeline development programs, demographic disparities in the various health sciences disciplines remain. As such the development of innovative interventions to help URM faculty and students overcome barriers to advancement remains a national priority. To date, the majority of pipeline development programs have focused on academic readiness, mentorship, and professional development. However, insights from the social sciences literature related to “extra-academic” (e.g., racism) barriers to URM persistence in higher education suggest the limitations of efforts exclusively focused on cognitively mediated endpoints. The purpose of this article is to synthesize findings from the social sciences literature that can inform the enhancement of URM pipeline development programs. Specifically, we highlight research related to the social, emotional, and contextual correlates of URM success in higher education including reducing social isolation, increasing engagement with research, bolstering persistence, enhancing mentoring models, and creating institutional change. Supporting URM’s success in the health sciences has implications for the development of a workforce with the capacity to understand and intervene on the drivers of health inequalities.

## Introduction

In the United States (US), the reduction of persistent health inequalities based on race and ethnicity remains a significant public health priority. The current landscape of infections, hospitalizations, and deaths associated with the coronavirus pandemic further underscores the urgency of addressing health inequalities in this country. To illustrate, in the past 10 months, more than 267,000 Americans have died as a result of COVID-19, the disease caused by the coronavirus. Of those, Blacks account for 22.9% of all COVID-19 deaths while representing only 13.4% of the total US population [1]. Many of these patterns are also observed in Latinx and Native American communities with COVID-19 disparities even more pronounced in cities and tribal areas with high concentrations of economic disadvantage [2]. Actions needed to halt the tide of the COVID-19 pandemic are immense and include the development and equitable distribution of effective vaccinations, improvement in access and quality of care, and active engagement and input from communities in public health education and prevention activities. Prior successes in curbing devastating epidemics (e.g., HIV/AIDS) have demonstrated the importance of addressing the specific socio-cultural needs of the groups most negatively impacted by the public health crisis. The disproportionate impact of the COVID-19 epidemic among US racial/ethnic minorities also requires culturally informed responses and underscores the urgent need to diversify not only the health care delivery workforce but also the biomedical and health sciences research workforce.

## Lack of Diversity in the Health Sciences

There is an ongoing and critical lack of diversity in the biomedical and health sciences workforce [3]. The inability to achieve workforce diversity goals in the health sciences has long been attributed to the failure of the academic “pipeline” to maintain a steady flow of under-represented minority students (URM; African American, American Indians/Alaska Natives, and Latinx populations) [4]. In response, the National Institutes of Health (NIH) and other entities across the biomedical and behavioral research landscape have committed considerable resources to increase the proportion of URM students in health science disciplines such as nursing, medicine, and pharmacy [5]. Nevertheless, the persistently low representation of URM students in the health sciences highlights institutional deficits in recruiting and retaining URM at all levels of the academic pipeline [4]. The failure to attract and retain URM students has implications for the overall quality of health sciences research and our ability to understand and intervene on the drivers of health inequalities in chronic and infectious diseases.

The development and dissemination of effective interventions to help URM students overcome barriers to enrollment and matriculation in health science disciplines remain a national priority. To date, the majority of pipeline development programs have focused on academic readiness, mentorship, and professional development [6]. However, a myriad of extra-academic factors (e.g., social, emotional, and contextual factors) have been linked to high attrition rates among URM students in higher education [7]. For example, findings from the US National Longitudinal Survey of Freshmen demonstrated that racial bias has a significant impact on attrition rates of URMs in science, technology, engineering, and math (STEM) and health science majors [8]. Further, McGee and Bentley [9] found that even among high-achieving URM students, experiences with racism caused URM students to question their abilities and value within STEM and health science majors. Despite the relevance, markedly less attention has been paid to addressing the extra-academic factors that hinder interest, performance, and retention of URM in STEM and health sciences fields.

The purpose of this article is to synthesize research findings from the social sciences literature that can inform the design of health sciences pipeline development programs for URM students and scholars. The sections below highlight research associated with social, emotional, and contextual factors deemed relevant for improving pipeline development programs including reducing social isolation, increasing engagement with research, bolstering persistence, enhancing mentoring, and creating institutional change. By highlighting the contributions from the social sciences, we seek to move beyond individual-level approaches that too often focus on the academic deficits of URM students and fail to address the complex nature of academic systems that maintain and reinforce inequalities.

## Reducing Social Isolation as a Means of Improving Pipeline Development Programs

The lack of representation of URM students and faculty in health-related majors and careers often leads to social and professional isolation. Compared to their non-Hispanic white counterparts, URM students at all levels of higher education and training often express higher rates of social isolation and distress associated with the psychosocial impact of “being the only one” [10]. Social isolation among URM students results from a lack of social support, insensitivity, and discrimination from peers and faculty [10]. Social isolation has been linked to feelings of marginalization, depression, anxiety, diminished self-esteem, low academic performance, and higher attrition rates of URM students in higher education [11].

To attenuate the experience of being the “only” or “one of a few” that URM students face in health sciences programs, it is imperative that recruitment and retention efforts reach a level of “critical mass” [12]. Studies have documented that when there is a significant representation of URM students and faculty at an institution of higher education, that is, a “critical mass,” URM students report an improved sense of support and inclusion [11]. It has been proposed that any racial/ethnic minority group needs to reach at least a 15% representation of the school population to attain critical mass [13]. To support efforts toward building a critical mass of URM students and faculty in the health sciences, we describe three strategies in the sections below: holistic review and admission processes, cohort admissions and cluster hiring, and inter-group contact and dialogue.

### Holistic Review and Admission Processes

A holistic review and admission process occurs when universities aim to assess the “whole” applicant inclusive of contextual factors that may inform their likelihood of program success [14]. Holistic review and admission processes have been proven effective in identifying the strengths and skills of URM applicants that may be overlooked in traditional review processes [14]. Specifically, holistic admission procedures weigh indicators of leadership abilities, persistence to achieving long-term goals, and community engagement that are outside of the traditional academic measures of success [14]. The ultimate desired outcomes of holistic reviews and admissions processes are to identify applicants who have the ability to succeed in the training program and who add to the diversity of backgrounds reflected in the student body. Research studies have shown that holistic admissions review practices increase the diversity of students without negatively impacting academic program success (i.e., graduate point averages and graduation rates) [15]. However, the key to the successful implementation of holistic admissions is that members of the selection committee be fully committed to the process. Onboarding and continuing education activities among all faculty, staff, and students involved in the admissions process are instrumental in maintaining fidelity to the principles of holistic admissions [15].

### Cohort Admissions and Cluster Hiring

Promoting a sense of community by creating a cohort of URM students with similar backgrounds can be effective in reducing social isolation [16]. One example of cohort admissions is the Posse Program [17]. In this program, the staff and mentors use non-traditional strategies to identify and train public high school students with strong leadership potential and send the students to college together as a “Posse.” The Posse Program has been shown to be successful in increasing the diversity and success of URM students that enter higher education [18]. Further, research also suggests that such cohort approaches can contribute to interdependent and mutually supportive relationships [26] and reduce feelings of anxiety resulting from social isolation [19]. Similar to cohort student admissions, cluster hiring of URM faculty is an initiative that seeks to increase the workforce diversity by hiring more than one URM faculty at the same time [20]. Cluster faculty hiring could be in the same department, inter-departmental, or across schools. Cluster hiring can help to minimize feelings of social isolation among URM faculty while promoting collaboration, social support, and peer mentorship. In turn, a higher representation of URM faculty can help to reduce feelings of social isolation, provide role models, and can increase graduation rates of URM students in those same institutions [21].

### Inter-group Contact and Dialogue

In addition to increasing the representation of URM students in higher education (i.e., the quantity of URM students), it is also important to encourage engagement among URM and non-URM students to improve the quality of the experience in the training program. The contact hypothesis [22] suggests that increasing inter-group contact by reducing social distance and encouraging inter-group communication is beneficial in reducing social isolation and facilitating effective interactions across diverse groups [23]. Inter-group dialogue is one approach for increasing knowledge, understanding, and social contact between students. Such dialogue seeks to facilitate collaboratively structured group conversation characterized by participants’ willingness to “listen for understanding.” This method has been employed frequently with student participants reporting increased self-reflection, awareness of self as a member of a social group, knowledge about structural group inequality, perspective-taking, and motivation and actions to bridge differences [24]. Additionally, Puchalska-Wasyl [25] found that inter-group contact reduced confrontational attitudes and made participants less inclined to gain an advantage over outgroup members, increased mutual openness to different viewpoints, and enhanced readiness to consider the arguments of others. By reducing social distance and encouraging inter-group contact, pipeline programs may be able to reduce social isolation experienced by URM students.

## Increasing Engagement with Research to Improve Pipeline Development Programs

Many health sciences training programs for URM students typically offer a combination of academic development opportunities such as social support, mentoring, stipends, tutoring and exam preparation, graduate school advising, summer bridge experiences, research opportunities, and career and skills development activities [6]. These learning opportunities increase academic skills and are necessary for successful advancement in the biomedical and health science fields. However, the benefits of these programs or increasing recruitment and retention of URM students have been limited [26]. Indeed, increased academic readiness alone may not be enough to engage many URM students in health sciences research. For example, many URM students who pursue higher education are often motivated by a desire to serve their communities [27]. Consequently, URM students with strong commitments to improving and serving communities may consider research-related careers, as traditionally presented, as esoteric and irrelevant to the solutions needed to reduce health inequalities.

Strategies to reshape the perceived relevance of research training and careers for URM are required in pipeline programs. These strategies can include the following: exploring the important contributions of URM faculty in community-engaged research, expanding theoretical approaches to critically understand the role of structural violence, racism, and other social determinants on health disparities, and expanding community-based service-learning (CBSL) research opportunities. The rationale for the inclusion of each of these topics into traditional pipeline development programs is discussed below.

### Highlighting the Contributions of URM Researchers

The contributions of URM researchers have expanded and enhanced our understanding of health disparities. URM researchers can bring about different perspectives concerning theory and practice, which effectively challenge existing views on health inequality. For example, Crooks et al., 2019 [28] has developed a framework, grounded in the sexual experiences of Black girls and women, to inform Black female sexual development and STI/HIV risk. Research guided by this framework has helped to identify socio-cultural conditions including the lack of protection (i.e., trauma-related factors and absence of parents due to systemic factors) and stereotype messaging that contribute to disproportionate sexual health disparities in this population. Crooks’ framework also has relevance to the development of effective and culturally tailored programming and interventions.

Despite the recognized importance of diversity in the health sciences, there is a lack of literature that focuses on the contributions of URM scholars [29] in reducing health inequalities. One study conducted by Bauer-Dantoin and Ritch [30] designed a class to examine the contributions of URM researchers in science. The course focused on the life histories of URM in science (i.e., Percy Julian) and the barriers they faced (i.e., racial discrimination and low SES). In addition, the class focused on the factors that helped URM in science overcome barriers to success [30]. Additionally, many URM researchers, often use qualitative methods including grounded theory, ethnography, community-based participatory research (CBPR) to elicit stories and experiences to better understand health disparities and participate in social justice [31]. Highlighting the importance of CBPR activities to increase community involvement can help to underscore the importance of community-informed research conducted by URM faculty in improving community health and promoting social justice [32].

### Expanded Theoretical Frameworks

Pipeline development programs in the health sciences should also seek to expand the training curriculum to focus on non-biological drivers of health inequalities. The extent to which individual behaviors are embedded in external context is now well documented in the social determinants of health literature [33], with the Social Determinants of Health Model [34] now a leading framework endorsed by the NIH. Additional theoretical frameworks have been developed to highlight the role of contextual factors such as structural violence and racism on health and health inequalities. For example, the minority stress theory emphasizes how external events such as discrimination can increase stress and negatively affect physical and mental health outcomes [35]. Geronimus’s Weathering hypothesis relates to Black women experiencing racism-related stress across the life course and that racism-related stress can lead to preterm births and low birth weight [36]. The Socioecological Model [37] incorporates multi-level social factors to guide health equity research. Critical race theory, which is grounded in social justice and race equity, encourages scholars to look beyond proximal factors of physical health and to consider housing, employment, and other social factors that affect health and well-being [38]. These theories can inform the type of research that URM students may be more interested in conducting and should be presented along with other established theoretical approaches.

### Service Learning Experiences

Lastly, the inclusion of community-based service-learning (CBSL) CBSL opportunities is recommended along with other traditional forms of research training. CBSL training gives students the opportunity to learn applied research skills. A growing body of literature suggests that CBSL provides a means by which the community and students mutually benefit through an exchange of knowledge [39]. CBSL is essential for practice-based disciplines such as public health, nursing, and medicine [39] and can have a long-term impact on under-resourced communities. CBSL can also be a useful means to learn about the importance of research interventions in “real world” community contexts. Additionally, CBSL can help bridge the gap and support partnerships between communities and universities.

## Bolstering Persistence as a Means for Improving Pipeline Development Programs

While there has been a range of institutional approaches aimed at increasing persistence among URM health science students, retention remains a significant problem. Early research on educational persistence among URM students placed a heavy emphasis on the academic disadvantage due to historical structural and systemic racism experienced by URM [40]. Growing evidence suggests that psychological factors also play an integral role in persistence among URM students. Minority stress is a salient predictor of psychological distress among students of stigmatized and marginalized minority groups [41]. Generally, students in health science programs are at increased levels of stress related to the rigorous nature of health disciplines [42]. However, URM students face unique minority stressors including discrimination, micro-aggression, and bias that are linked to poor academic and social integration [43]. Stereotype threat and internalized bias have been associated with racial gaps in academic performance [43].

Training curriculums should include information about these internalized barriers to the advancement of non-traditional students in the health sciences including women, URM, and first-generation students. In addition, resiliency frameworks, rather than deficit models, should guide strengthening URM student persistence interventions [44]. Resilience models aim to cultivate resilience skills or “grit” among URM students [45]. Although these innovative programs hold promise for cultivating persistence among URM students, evaluation data are limited. Further research is needed to evaluate the strength of multi-dimensional interventions to cultivate socio-emotional and psychological well-being of URM students in health science programs to improve persistence.

## Enhancing Mentorship as a Means for Improving Pipeline Development Programs

Mentorship as it relates to gaining knowledge about academic culture, developing research skills, teaching, and service, and overall career advancement is essential to the success of URM students and scholars in higher education [46]. Although the importance of mentoring is highly recognized for student and faculty success, there are prevailing challenges in mentoring URM scholars. Effective mentors can forge meaningful connections, provide scholarly opportunities, offer critiques, advise on academic politics, and help the mentee focus [47]. However, it has been shown that URM students often have difficulty securing mentors. Zambrana and colleagues [47] identified the following barriers to mentorship: the lack of social capital, limited URM mentors, and undervalued URM faculty’s scholarship. To address these barriers, it is important to consider the characteristics of the mentoring relationship. For example, some scholars argue that mentorship should provide both instrumental and social support to achieve successful mentorship for URM students and scholars [48]. Additionally, departmental commitment to formal mentorship activities of URM faculty should be part of organizational practice. Formal mentoring relationships are important for career development and social support to address the challenges that URM scholars experience in building professional networks and countering institutional barriers [49].

## Creating Institutional Change as a Means for Improving Pipeline Development Programs

Institutional level policies are critical to improving the URM pipeline in higher education. While the need for institutional level climate change has been documented [50], efforts to change institutional norms as to what is valued in teaching, research, and service warrant further development. Further, the success of URM students and faculty needs to become an important institutional goal. We review two perspectives underpinning institutional changes for successful URM pipeline programs: social closure and detracking. These approaches are important because they provide guiding foundations for institutional reforms for diversity in higher education at all levels.

### Social Closure Perspective

The issue with the “leaky pipeline” indicates URM scholars are less likely to enter into higher education academic careers, which then continues to be “leaky” at all stages of academic education, training, promotion, and leadership development. One of the frameworks explaining the causes of such leaky pipeline is the idea that the practices of already privileged groups promote maintaining their status and limiting opportunities for outsiders. This process of drawing boundaries and constructing identities around the social boundaries is called social closure. The main purpose of social closure is to control resources.

Concerning racial/ethnic inequality in higher education, social closure is a mechanism through which URM scholars are often excluded from resources and opportunities relations. Practices of closure could work in evaluation, resource distribution, and promotion. In particular, evaluation often works against URM scholars). While the academy is viewed as an institution of meritocracy, organizational decisions are influenced by implicit biases and stereotypes against minority scholars, which disadvantages URM in hiring and promotion. Social closure also limits access to resources and opportunities. This “opportunity hoarding” ensures the members of the privileged monopolizes resources and opportunities, while excluding the other. In the academy, URMs often experience limited access to resources, know-hows, and social networks that are critical to successful socialization and promotion due to implicit and explicit practices of social closure.

These discriminatory practices become institutionalized, in part because organizational leadership often lacks minority representation, thus privileged preferences and stereotypes are reflected in URM evaluation, resource distribution, and social relations including mentorship and training opportunities. At the same time, because social closure prevents URMs from leadership roles, such practices are not challenged.

### Detracking

Detracking in education is an argument against the practice of grouping students based on their academic ability levels, instead, detracking aims to create mixed classes with students of different abilities. In the tracking system, teacher’s expectations often differ for high track and low-track students. Furthermore, more resources are directed to high-track classes, while low-track classes tend to be low-income disadvantaged students in the first place, resulting in wider gaps between high and low performing students. Such a segregationist model of education may not reflect how students learn and interact with others. Detracking also challenges how people think about intelligence and stereotypes about race.

## Conclusions

In this article, we described concepts and strategies from the social sciences that may help to increase the overall effectiveness of pipeline development programs for URM students in health sciences disciplines. We have summarized several key areas of research that go beyond the traditional focus on academic endpoints that may serve to increase the effectiveness of pipeline development programs for the health sciences. Future research is needed that evaluates the added benefits of pipeline development programs that address the combined academic, social, emotional, and environmental barriers to academic success.
